# Assessing Students’ Critical Thinking in Dialogue

**DOI:** 10.3390/jintelligence12110106

**Published:** 2024-10-26

**Authors:** Ruiguo Cui, Lili Zhao

**Affiliations:** 1 School of Foreign Languages, Taizhou University, Taizhou 318000, China; cuiruiguo97@tzc.edu.cn; 2 School of Business, Taizhou University, Taizhou 318000, China

**Keywords:** critical thinking, assessing, qualitative, dialogue

## Abstract

Critical thinking has been widely considered an important skill in the 21st century. In view of the value attached to critical thinking, various quantitative instruments have been developed to assess critical thinking, which only provide a product of critical thinking and cannot reveal the critical thinking process of test takers. Hence, this paper proposes a coding scheme facilitating a qualitative analysis of critical thinking exhibited in interaction. The coding scheme consists of five categories of critical thinking skills, i.e., analysis, comparison, evaluation, inference, and synthesis, each of which is coded at low, medium, and high levels. The use of this coding scheme is then illustrated by applying it to authentic classroom dialogue. This coding scheme is hopefully conducive to the assessment of critical thinking in educational settings.

## 1. Introduction

Critical thinking has been widely considered as an important skill for success in learning, working, and life in the 21st century (Angeli and Valanides 2009; Halpern 2014; Paul 1993; Rear 2019; Teo 2019). Characterized by a list of skills and dispositions (Ennis 1987), critical thinking helps individuals avoid being egocentric (Wright 2002), enables individuals to think independently and guard against being manipulated (Vieira et al. 2011), and mediates the relationship between essential skills, such as algorithmic thinking, creativity, digital literacy, and effective communication, and problem solving (Kocak et al. 2021). Furthermore, critical thinking is related to ethics. A critical thinker will not confine his thinking only to his own interests but consider the interests of all related persons (Paul 1993). Critical thinking is arguably more and more important with the advances in online technologies such as ChatGPT (Dumitru and Halpern 2023).

Given all these values attached to critical thinking, it has seized significant and sustained scholarly attention. The intellectual interest in critical thinking can be traced back to Socrates, who “ask[ed] probing questions in an effort to expose the values and beliefs which frame and support the thoughts and statements of the participants in the inquiry” (Reith 2003, p. 1). This critical spirit was inherited and passed down to scholars over the generations such as Plato, Kant, Marx, and Dewey (Coney 2015; Vieira et al. 2011). Though Dewey is widely recognized as the father of modern critical thinking with his concept of ‘reflective thinking’ (Fisher 2011; Sternberg 1986), it was Edward Glaser who stoked the academic enthusiasm for critical thinking with his ‘Experiment in the Development of Critical Thinking’ (Glaser 1941; Paul 1985, 1993). This renewed interest in critical thinking culminated in the so-called ‘critical thinking movement’ (Paul 1985) in the 1980s and the inclusion of critical thinking in 21st century skills (Hilliker and Loranc 2022; Partnership for 21st Century Skills 2006; Taar and Palojoki 2022).

In line with this intense interest in critical thinking, educational researchers and practitioners have been proposing and experimenting with ways of developing students’ critical thinking in the classroom. For example, Mercer and his colleagues have proposed the practice of ‘thinking together’ to promote students’ critical thinking in ‘exploratory talk’ (Mercer 1995). In contrast to ‘disputational talk’ (in which students only talk to dispute with each other without making constructive contributions) and ‘cumulative talk’ (in which students uptake others’ contributions without any critique), exploratory talk encourages students to engage in constructive critique of one another’s ideas and thus engages students in collective thinking and critical thinking (Mercer 1995; Mercer and Littleton 2007). Other scholars have also agreed that classroom dialogue is a viable avenue for students to practice and develop critical thinking (Alexander 2006; Barnett and Francis 2012; Brookfield 2011).

Considering the value of dialogue in the development of critical thinking, there is a pressing need for the evaluation of critical thinking in dialogue. Currently, such assessment tools are scarce in spite of a myriad of standardized tests for the purpose of education and recruitment, such as the Watson-Glaser Critical Thinking Appraisal (Watson and Glaser 1980), the California Critical Thinking Skills Test (Facione et al. 1998), the Cornell Critical Thinking Tests (Ennis et al. 1985), the Halpern Critical Thinking Assessment (Halpern 2007), and the HEIghten Critical Thinking Assessment (Liu et al. 2018). Moreover, these standardized tests only provide a product of critical thinking and cannot give us a glimpse of the critical thinking process of test takers. There are also some concerns about the validity of such standardized tests as critical thinking is sometimes viewed beyond precise quantification and measurement (Frisby 1991) and about their reliability since some test takers can fake on these tests by answering in a way that does not reflect their true opinion but makes them seem like a critical thinker (Silva 2009).

Hence, this paper proposes a coding scheme of critical thinking, initially developed for the analysis of classroom dialogue yet potentially useful for analyzing qualitative data in many other contexts, such as dialogues in everyday life.

## 2. Conceptualization of Critical Thinking

Although critical thinking has been discussed for centuries and has been gaining traction in the current educational landscape across the globe, there is little consensus over the definition of this multifaceted and elusive construct. Numerous definitions have been given to the term critical thinking. For example, McPeck (1981) suggested that “critical thinking is the appropriate use of reflective scepticism, and that this is necessarily linked with specific areas of expertise and knowledge” (p. 19). Ennis (1987) defined critical thinking as “reasonable reflective thinking that is focused on deciding what to believe or do” (p. 10). Paul and Elder (2012) defined critical thinking as “thinking explicitly aimed at well-founded judgment, using appropriate evaluative standards in an attempt to determine the true worth, merit, or value of something” (p. xxv). Halpern (2014) defined critical thinking as “the use of those cognitive skills or strategies that increase the probability of a desirable outcome…in solving problems, formulating inferences, calculating likelihoods, and making decisions” (p. 8). To Dumitru and Halpern (2023), critical thinking “encompasses intellectual skills such as reflection, self-regulation, analysis, inference, explanation, synthesis, and systematic thought” (p. 3).

There is also a lack of consensus on the nature of critical thinking. Some (e.g., Petress 2004; Vaughn 2005) believe that critical thinking involves following certain procedures and satisfying some criteria. Lipman (1988), for example, thinks that critical thinking is “skillful, responsible thinking that facilitates good judgment because it (1) relies upon criteria, (2) is self-correcting, and (3) is sensitive to context” (p. 39). Others view critical thinking as more a form of ‘knowing how’ than a form of ‘knowing that’, more “a possession of certain skills” (Mulnix 2012, p. 468) than a possession of certain knowledge. This is why Paul (1990) disagreed when McPeck (1990) argued that training of critical thinking skills does not work since critical thinking needs subject knowledge and therefore cannot be transferred across disciplines. This is echoed by Rickles et al. (2013), who believe that “critical thinking can be conceived of as a skill or a process, rather than a body of knowledge” (p. 272). However, Willingham (2007) pointed out that “critical thinking does not have certain characteristics normally associated with skills, in particular being able to use that skill at any time” (p. 15). He explained that a new skill such as reading music is available for use at any time after it has been learned, but critical thinking is not the case. Even after extensive training, people may still fail to become critical thinkers.

Such disagreements are due partly to the fact that discussions about critical thinking are rooted in different disciplines. There are three main disciplines in which critical thinking has been conceptualized, i.e., philosophy, psychology, and education (E. Lai 2011). The philosophical approach focuses on the qualities of ideal critical thinkers and their thoughts; the cognitive psychological approach focuses on the behaviors of critical thinkers and procedures of critical thinking; and the educational approach focuses on fostering learners’ skills necessary in solving problems and making decisions (E. Lai 2011; Sternberg 1986).

This lack of consensus on the conceptualization of critical thinking is also a result of three different perspectives towards the nature of critical thinking as trait, emergent, and state (Halonen 1995). Researchers adopting a trait perspective interpret critical thinking as an inherent disposition to think critically; the emergent perspective suggests that children’s critical thinking abilities emerge naturally during their interaction with the environment; and the state perspective explicitly regards critical thinking as a set of skills and abilities (Halonen 1995). Though different in nature, these three perspectives of critical thinking seem not to be mutually exclusive but complementary, as they stress different aspects of critical thinking. The trait perspective focuses on the trait or disposition of critical thinking, while the emergent and state perspectives focus on the abilities or skills of critical thinking. The abilities of critical thinking may be latent or manifest, which are explained by the emergent perspective and the state perspective, respectively. The latent critical thinking abilities refer to individuals’ potential for critical thinking as a result of natural development, which relies little on instruction, while the manifest ones refer to individuals’ abilities to employ and exhibit their latent critical thinking abilities, which may develop as a result of instruction.

Therefore, at the heart of the three perspectives on the nature of critical thinking is the differentiation between critical thinking disposition and critical thinking abilities. According to Watson and Glaser (1980), critical thinking is “a composite of attitudes, knowledge, and skills” (p. 1). Many other scholars also agree that critical thinking is composed of both disposition and skills (Ennis 1987; Facione 2000; Paul and Elder 1997). Hence, critical thinking arguably has a dual nature, one that is relatively static and the other more dynamic: (1) the disposition of critical thinking that is relatively stable and less susceptible to change; (2) the abilities of critical thinking that are susceptible to change as a consequence of environmental impact or formal instruction.

Recently, there is a holistic and integrated view in which critical thinking is a composite of skills, dispositions, and action in disciplinary contexts (Yuan and Liao 2023). Conceptualized in this way, critical thinking is not merely limited to certain skills and dispositions but “embraces an action orientation with an overriding focus on changes and transformation within individuals’ situated realities” (Yuan and Liao 2023, p. 545). Therefore, critical thinking is closely linked to critical pedagogy, and the goal of critical thinking is not just cognitive enhancement but social change.

## 3. Assessment of Critical Thinking

Various tools have been developed to assess critical thinking (Butler 2024; Fisher and Scriven 1997; Liu et al. 2018). Some, such as the California Critical Thinking Disposition Inventory (Facione et al. 2001), aim to assess critical thinking disposition, that is, to test a person’s tendency to think critically, while others are used to assess critical thinking abilities, that is, to test a person’s actual performance or application of critical thinking.

The Watson-Glaser Critical Thinking Appraisal (WGCTA) is perhaps one of the most widely used examples of the latter. Using 80 items, or 40 items in the short form, the WGCTA assesses five critical thinking abilities: making inference, recognizing assumptions, making deduction, interpreting arguments, and evaluating arguments (Watson and Glaser 1980). In the WGCTA, examinees are asked to read a number of scenarios of statements, arguments, or problems, each of which is followed by a list of items in the form of inferences, assumptions, deductions, conclusions, and arguments. The examinees have to make a specific judgment of these items on the basis of each scenario, and these judgments are then scored so as to numerically gauge their five critical thinking abilities. A similar standardized measurement is the California Critical Thinking Skills Test, which assesses critical thinking on six scales: analysis, evaluation, inference, deduction, induction, and overall reasoning skills (Facione et al. 1998). By generating a score, these assessment tools are useful for quantitatively measuring and ranking critical thinking abilities. Furthermore, these instruments are not context-dependent and can therefore be applied in various contexts.

However, a number of researchers harbor reservations about the use of such quantitative instruments in educational establishments due to several reasons. First, these instruments do not consider contextual factors such as classroom conditions, which may compromise their validity (Norris and Ennis 1989). Second, they only measure the product of critical thinking and pay little attention to the process of critical thinking (Norris 1985). In other words, a score yielded by these quantitative instruments only indicates whether or to what degree a person thinks critically, but not how a person thinks critically. Third, they fail to detect students’ growth or development in critical thinking abilities (McMillan 1987).

In view of these limitations of tools that focus exclusively on the product of critical thinking, a more qualitative approach to the assessment of critical thinking is sometimes preferred. The authenticity and depth of qualitative data may entail creating a naturalistic and information-rich environment that is conducive to studying the manifestation of critical thinking and hence allows researchers to gain more insights into the complex nature of critical thinking.

There are different forms of qualitative assessment available in educational settings. White et al. (2011) used open-response questions asking students to handle, interpret, and analyze a set of complex and conflicting data so as to test science students’ critical thinking abilities to deal with conflicting data, resolve research ambiguity, and conjecture different interpretations of the same data. Argumentative writing (Stapleton 2001) and online discussion (K. Lai 2012) were also used to assess students’ critical thinking. For instance, Stapleton (2001) asked students to write argumentatively in response to a provocative essay, and these writings were then judged in terms of critical thinking based on five elements: arguments, evidence, opposite viewpoints, refutations, and fallacies. K. Lai (2012) utilized online discussion to evaluate her students’ critical thinking by asking her students to discuss a selected text and then assessing these discussions based on certain criteria of critical thinking skills. Tsui (1998) also suggested that classroom observation, which has been largely neglected, should be adopted since this qualitative method enables a researcher to examine and evaluate the process of students’ critical thinking.

Few methods have been proposed yet to systematically analyze students’ critical thinking exhibited in classroom interaction, which is a fitting venue for the observation and development of students’ critical thinking. One exception can be found in Fernandes et al. (2024), who developed an analytical framework of critical thinking to evaluate the effects of a teacher’s facilitation in an English listening and speaking classroom. This analytical framework focuses on five types of students’ practices that are believed to facilitate the development of students’ critical thinking, i.e., to evaluate and determine the credibility of given information, to find and generate key ideas from given information, to embark and clarify key ideas, to organize and manage given information, and to analyze and synthesize given information. Each of the five practices will be evaluated in terms of its clarity, relevance, depth, and coherence. However, this analytical framework does not attempt to differentiate the levels of students’ critical thinking. Hence, our purpose in this research is to propose a coding scheme to facilitate a qualitative assessment of critical thinking abilities in dialogue.

## 4. A Coding Scheme of Critical Thinking

In spite of some coding schemes for analyzing dialogue, few are specifically devoted to critical thinking. For example, the Conversational Argument Coding Scheme developed by Seibold and Meyers (2007) is mainly focused on argument, while Multiple Episode Protocol Analysis (MEPA) developed by Erkens (2005) is used to code dialogue acts. Hence, our study is aimed at developing a coding scheme specifically targeted at critical thinking. This coding scheme includes five skills of critical thinking, i.e., analysis, comparison, evaluation, inference, and synthesis, which have been widely acknowledged as being the most important critical thinking skills (e.g., Coon and Mitterer 2010; Dwyer et al. 2014; Ennis 1987; Facione 1990). Among these five skills, analysis, inference, and evaluation are regarded as the core skills of critical thinking (Dwyer et al. 2014; Facione 1990). Paul and Elder (2012) also agree that critical thinking is analytical, inferential, and evaluative thinking. In particular, evaluation is at the heart of critical thinking, and it is not an overstatement to say that critical thinking is evaluative thinking (Facione 1990; Yinger 1980; Young 1980). Critical thinkers evaluate not only other persons’ ideas and thoughts but also their own thinking. This means that the skill of evaluation has an aspect of reflection, which aligns with Ennis’ (1987) definition of critical thinking as “reasonable reflective thinking that is focused on deciding what to believe or do” (p. 10). Comparison is also an important critical thinking skill, as a critical thinker will weigh up the advantages and disadvantages of an idea or issue and compare multiple perspectives and ideas (Moon 2008; Phillips and Bond 2004). Synthesis is important considering that critical thinking is “an orientation to transform learning and society” (Benesch 1993, p. 546). That is, critical thinkers not only critique others’ ideas but also provide an alternative. This alternative is not created solely based on critical thinkers’ thoughts but a result of collective intelligence/thinking. It is building on others ’thinking, or an extension of others’ thoughts. In other words, critical thinkers synthesize others’ ideas based on their evaluation and the available information (Jacobs et al. 1997).

Some models of critical thinking (e.g., Facione 1990) also regard mental activities such as understanding, comprehension, and interpretation as a sub-skill of critical thinking. It is true that critical thinking would be like a castle in the air without these basic skills. If these basic skills were included, however, critical thinking would be arguably equivalent to thinking in a general sense (Dwyer et al. 2014). Thus, basic skills such as understanding, comprehension, and interpretation were not included in this coding scheme for critical thinking. In this coding scheme (see Table 1), each skill can be manifested in student talk on three levels, that is, low level, medium level, and high level.

When using this coding scheme to code student talk in terms of critical thinking, the coder should first of all examine whether the students have made an attempt at critical thinking so as to distinguish student talk with critical thinking from student talk with no critical thinking. That is, if students display an awareness of critical thinking and attempt to analyze, compare, evaluate, infer, or synthesize, their talk would be deemed to have demonstrated critical thinking. For student talk with a display of any of the five critical thinking skills, the coder would then evaluate the level of these skills.

When deciding the level of critical thinking displayed in student talk, the factor of depth is taken into consideration. It is concerned with whether a student’s critical thinking is superficial or sophisticated by reference to the logic and the contextual information.

## 5. Applying This Coding Scheme to Authentic Student Talk

In order to assist the understanding of this coding scheme and better illustrate its application in specific classroom dialogue, the five skills of critical thinking, i.e., analysis, comparison, evaluation, inference, and synthesis, are defined in reference to the various literature, such as Dwyer et al. (2014), Ennis (1987), and Facione (1990). Following that, excerpts of authentic classroom dialogue are taken from a large project on Chinese college students’ English classroom talk to exemplify the analysis of the five skills of critical thinking exhibited in student talk. In this English classroom, these Chinese students, around the age of 18, participated in English dialogues based on some texts on certain topics in order to improve their English proficiency as well as their thinking skills.

The unit of analysis when coding students’ critical thinking displayed in these classroom excerpts is the individual discourse move “defined as a single utterance or a string of uninterrupted utterances with a common function”, which is commonly employed as the unit of analysis in the study of classroom discourse (Lefstein et al. 2015, p. 870).

### 5.1. Analysis Exhibited in Student Talk

Analysis refers to the ability to break down information, issues, opinions, ideas, or arguments into their organic constituent elements or to identify/establish the relationships among information, issues, opinions, ideas, or arguments (Dwyer et al. 2014; Ennis 1987; Facione 1990).

Low-level analysis can be found in Excerpt 1, when the teacher asked students to come up with some important skills in the 21st century.

Excerpt 1
TurnSpeaker
Code1T:Anything else [that is important in the 21st century]? 


(Silence for 9 s)
2Si Mei:Reading skill.
3T:Reading skill. Why do you think reading skill is important?
4Si Mei:People need reading skill not only reading books but also reading people’s mind.Al

In this excerpt, Si Mei demonstrated her skill of analysis when asked to explain the importance of reading skill. It can be noticed from her reply in Turn 4 that Si Mei first of all broke the issue of reading skill into different constituent parts, i.e., reading books and reading minds, and emphasized the part of ‘reading people’s minds’ by means of the syntactic structure ‘not only… but also…’. Thus, Si Mei’s talk in Turn 4 revealed her effort to make an analysis of the importance of reading skills. However, such analysis was not deep since Si Mei did not elaborate on the role of reading skill in reading books and people’s minds and failed to focus her explanation on the specific role of reading skill in the 21st century. She could have raised the level of her analysis if she had pointed out why reading minds was needed for people and why such a skill was especially important in the 21st century. With such elaboration, a clearer relationship between reading skill and the 21st century would be established, and thus a higher level of critical thinking in terms of analysis would be displayed.

In comparison, Excerpt 2 provides an example of students’ analysis at a higher level.

Excerpt 2
TurnSpeaker
Code1T:What are you afraid of?
2He Wei:I’m afraid of talk face to face with the opposite sex. The reasons are the following. Firstly, I’m very shy. I don’t like talk with others, especially the opposite sex. I have little chance to talk with girls from childhood. Secondly, I lack confidence. The ways of overcoming these are as follow. First, I should talk with the opposite sex more. Second, I should lift up my confidence. Last but not the least, I should take part in many events to talk with people.Am

In this excerpt, He Wei demonstrated his skill of analysis at a high level by breaking down the issue of fear of talking with girls. In his analysis, he identified the reasons for such fear and solutions to overcome it by establishing causal relationships between the issue of fear of talking with girls and the factors that he had argued. In other words, he first of all separated the issue of his fear of talking with girls into its constituent parts and focused to scrutinize its reasons and solutions. Then he related such fear to various factors, among which shyness, diffidence, and little practice were identified as factors attributing to his fear of talking with girls. His analysis was deep in terms of his elaboration on these factors and clearly expressed in terms of his use of such words as ‘reasons’ and ‘ways’ as well as the discourse markers such as ‘firstly’ and ‘secondly’. However, He Wei’s analysis still has some problems. For example, the reasons he offered were not specifically for the fear of talking with girls but for the fear of talking with people. Therefore, He Wei’s talk in Turn 2 displayed his skill of analysis at a medium level.

### 5.2. Comparison Exhibited in Student Talk

Comparison is the ability to identify similarities or differences between different information, issues, opinions, ideas, or arguments (Ennis 1987; Facione 1990). Excerpt 3 is an example of students’ comparison at a low level. Based on a text about traveling solo, the talk in this excerpt followed Li Jun’s interpretation of the meaning of Paragraph 7 in this text.

Excerpt 3
TurnSpeaker
Code1T:OK. According to you and your group members, the author [in Paragraph 7] wants to demonstrate that culture sometimes is able to be reflected from the way they made contact with others. Right?
2Li Jun: Yes.
3T:OK.
4Li Jun:Paragraph 9, I think the meaning is the same with Paragraph 7 because I think different place has different understanding. And maybe the people in the Italy think they take their serving for granted, but in China the local people think they want to dedication. So I think the meaning is same with Paragraph 7Cl

In Turn 4, Li Jun made an attempt to point out the similarity between Paragraph 7 and Paragraph 9 in terms of their meaning. Such an attempt at comparison was especially commendable considering that Li Jun spontaneously made such a comparison between the two paragraphs. Although only asked to interpret the meaning of Paragraph 7, Li Jun went a step further to compare it with Paragraph 9, probably trying to validate his interpretation of Paragraph 7. However, despite his effort, Li Jun’s comparison in Turn 4 was not deep since he made no attempt to identify any differences between the two paragraphs. Furthermore, his comparison was not clearly expressed since it was difficult to understand from his comparison the meaning of Paragraph 9 and the similarities between the meaning of Paragraph 7 and Paragraph 9. Hence, Li Jun’s comparison displayed in Turn 4 was only at a low level.

Excerpt 4 is an example of a medium-level comparison in which Wang Chen was asked whether physical injury or psychological trauma sustained in a war is more severe.

Excerpt 4
TurnSpeaker
Code1Wang Chen:We think psychological trauma is more damage than physical injury. Because physical injury can be cured but psychological trauma never disappears. And psychological trauma can cause physical injury. They may hurt themselves when they suffer the pains from the war. And psychological trauma may have bad influence on their family and friends who are familiar with them. When a war breaks out, it will bring the fear to the people. So we think the psychological trauma is more damage than physical injury.Cm2T:OK. Good, very good. I totally agree with one sentence you have mentioned. Psychological trauma can also lead to physical injury. They may hurt themselves, they may hurt others. Right? They may hurt others. Since there are so many cases in the real life, there is no need for us to give any example to demonstrate.


In Turn 1, Wang Chen made a deep comparison between physical injury and psychological trauma caused by war by identifying the differences between the two kinds of war damage on three aspects. The first aspect was the curability. She asserted that physical injury can be cured but psychological trauma cannot. The second aspect she compared was their mutual relationship. Wang Chen pointed out that psychological trauma can result in physical injury since victims of psychological trauma may hurt themselves. Third, she compared their influence on other people. She highlighted the negative influence of a person’s psychological trauma on their family and friends.

The depth of Wang Chen’s comparison is reflected not only in the number of differences between physical injury and psychological trauma but also in the significance of these differences. The differences in the three aspects are critical rather than trivial between the two kinds of war damage. According to Wang Chen, there is a causal relationship between psychological trauma and physical injury. Since psychological trauma can cause physical injury, it is a more severe damage compared to physical injury. The third aspect of comparison (negative influence on others) reflects the potential damage of psychological trauma and physical injury on society.

Though Wang Chen’s comparison is deep, it still has some problems. When comparing the curability of the two types of war damage, Wang Chen was not persuasive to say that physical injury can be cured while psychological trauma cannot. Such a statement is not applicable in all cases since some physical injuries, such as loss of limbs, are permanent, while some psychological trauma can be overcome with therapy and counseling over time. In such cases, it may be inaccurate to say that psychological trauma is more damaging than physical injury. It is also fallacious to assume that the causal relationship between psychological trauma and physical injury is unidirectional. Based solely on the common life experience, ‘once bitten, twice shy’ for example, it is not difficult to realize that physical injury can result in psychological trauma as much as the other way around. In other words, psychological trauma and physical injury go together in certain cases. In this regard, it is difficult to say from this comparison which one of them is more severe. As for the third aspect of her comparison, Wang Chen also was not accurate to emphasize the negative influence of psychological trauma on other people while ignoring the negative influence of physical injury. Both psychological trauma and physical injury of war victims can negatively influence people around them, subjecting others to unpleasant or even dangerous situations. Thus, comparing the two types of war damage in this way cannot help one decide which one is more harmful. From the above analysis, it can be seen that Wang Chen’s comparison was only at a medium level.

### 5.3. Evaluation Exhibited in Student Talk

Evaluation, a skill sitting at the heart of critical thinking (Yinger 1980; Young 1980), refers to the ability to judge the credibility, validity, value, or significance of information, issues, opinions, ideas, or arguments (Dwyer et al. 2014; Ennis 1987; Facione 1990).

The following excerpt is an example that demonstrates students’ low-level evaluation. This excerpt was situated in a whole-class discussion of various characteristics of a job, such as job pay, job location, and the prospect of promotion. The discussion was preceded by a pair interview in which two students interviewed each other to find out which job characteristic was the most important factor to consider when seeking a job. After the pair interview, the teacher asked some students to share what they had found out from their interview. For example, before this excerpt, a student, Meng Jia, shared his choice of pay as the most important factor to consider during his job selection. His rationale for such a choice was that he needs money to support his family since he, as a man, should be the breadwinner in his family. Excerpt 5 started with the teacher’s elicitation of Sui Rui’s own attitudes towards those job characteristics.

Excerpt 5
TurnSpeaker
Code1T:OK. How about yourself?
2Su Rui:I think the job pay and responsibility is the most important because I want to make my finance independent.El3T: Financial situation.
4Su Rui:Financial independence. For example, he thinks, I think woman could be independent in the family, so… (Here ‘he’ refers to Meng Jia who said earlier that men should earn money to support the family.)El5T:(interrupting Su Rui) You don’t agree with him.
6Su Rui:Yeah. I don’t agree with him. If you have more responsibility, you have to be more seriously and maybe more stress. If I have the responsibility, I will try my best. If it’s beyond my responsibility, I will not. I will have too much stress.El

In this excerpt, Su Rui was asked about the most important job characteristic to her after she shared the choice of her interviewee. Her reply demonstrated her skill of evaluation in two instances. In the first instance, Turn 2, Su Rui deemed pay and responsibility to be the most important factors when seeking a job, and she also tried to justify such an evaluation. In another instance in Turn 4, Su Rui exhibited this skill by evaluating Meng Jia’s opinion. In this turn, she argued that women could also be financially independent in their family if their pay was good, and therefore Meng Jia’s opinion that men should be the primary, if not the sole, bread earner for their family was wrong.

Despite Sui Rui’s attempt to evaluate, her evaluation was not deep and well supported. Her evaluation of the significance of pay and responsibility in Turn 2 was not supported by her statement that she wanted to be financially independent, which shows that the evaluation exhibited in Turn 2 was only at a low level. In Turn 4, Su Rui’s evaluation of Meng Jia’s opinion was also at a low level since her evaluation was not well supported. As can be seen in this turn, Su Rui’s rationale for her evaluation of Meng Jia’s opinion was that women could be financially independent with a well-paid job. This can be put in the form of an argument, that is, Meng Jia was wrong to say that men should be the bread earner in the family because women could be financially independent with a well-paying job. In this argument, the reason seems not sufficient to arrive at the conclusion since Meng Jia was talking about men’s (and hence suggesting women’s) responsibility of earning money while Su Rui was talking about women’s ability to earn money.

The disconnect between the reason and the conclusion in Su Rui’s argument might not be a result of her lack of reasoning but rather a result of her neglect of repairing her reasoning chain. A closer look reveals that Su Rui was interrupted by the teacher in Turn 4 before aligning herself with what the teacher elicited in Turn 5, that is, the evaluation of Meng Jia’s opinion. Su Rui explicitly expressed her evaluation, or her disagreement in this instance, only after the teacher’s elicitation for clarification in Turn 5. In other words, Su Rui’s evaluation in Turn 6 might not be what she initially wanted to say. She did attempt to make a conclusion in Turn 4, which was indicated by the reasoning word ‘so’, before she was interrupted by the teacher. After the sidetrack, Su Rui did not justify her evaluation or resume the chain of her reasoning about pay and financial independence. Consequently, her claim of her disagreement with Meng Jia was left without solid support.

Students’ low-level evaluation can also be illustrated in Excerpt 6, a conversation between the teacher and a student with regard to an idea proposed by other students.

Excerpt 6
TurnSpeaker
Code1T:As this group and that group have mentioned, they are afraid of snakes. So they try to get more exposure to snakes. Is it a good method of overcoming fear? If you are afraid of something, you should try to get more exposure to it. Is it useful?
2Xue Hua:I think it is not useful. If you are afraid of snakes, it is bad to play with snakes. Sometimes you will feel more fear than before.El

In Turn 2, Xue Hua made an evaluation that the idea of overcoming fear of something by means of more exposure to it was not valid. She also tried to justify her evaluation by pointing out the danger of this idea. Although Xue Hua demonstrated her attempt to evaluate, her evaluation was not well justified. Since being exposed to snakes can take many forms, such as observing them from a safe distance in a zoo or even watching educational documentaries, it is not necessarily ‘playing with snakes’ as discussed by Xue Hua. Therefore, Xue Hua’s argument about the danger of playing with snakes cannot justify her evaluation that overcoming fear of something by means of more exposure to it is not useful.

### 5.4. Inference Exhibited in Student Talk

Inference is another core critical thinking skill, which means the ability to draw logical conclusions from information, observation, experience, judgment, theory, or hypothesis (Dwyer et al. 2014; Ennis 1987; Facione 1990).

In Excerpt 7, when asked about the relationship between Steven Spielberg and his father, a student made a low-level inference based on the information provided in the text.

Excerpt 7
TurnSpeaker
Code1TWhat is the relationship between Steven Spielberg and his father like?
2 Lin Shan:For he and his father, sometimes he would miss his father when he left home, but when he returned home, he would again furiously argue with his father. I think their relation is a little bad.Il

Based on the information in the text that Steven argued with his father when at home but would miss him when Steven was away from home, Lin Shan inferred that their relationship was ‘a little bad’. This demonstrated Lin Shan’s attempt to reach a conclusion based only on partial information in the text. However, since Lin Shan did not elaborate on her inference, it was not clear how she drew such a conclusion. Specifically, when inferring that their relation was ‘a little bad’, Lin Shan emphasized the fact that Steven argued with his father when at home, ignoring another fact that he would miss his father when leaving home. Thus, the inference made by Lin Shan in this excerpt was not deep and thus at a low level.

This low-level inference could be elevated to a higher level if Lin Shan had elaborated her inference to some extent. One possible way is for Lin Shan first of all to concede Steven’s love towards his father before highlighting the tension between the two. In doing so, she would demonstrate that her inference was based on all available information and not just partial information. Then she could explain her reasoning process so as to lend more support to her inference. For example, she could have referred to the fact that the arguments between Steven and his father were not occasional but regular and argued that this would not happen if they enjoyed a good relationship. Such elaboration would make her inference more logical and deeper, thus elevating it to a higher level.

Low-level inference can also be illustrated in the following dialogue, which transpired when the whole class brainstormed important factors for a person’s success.

Excerpt 8
TurnSpeaker
Code1T:Anything else [that is important for a person’s success]?
2Gu Lan:Interest.
3T:Interest. Good. Interest.
4Gu Lan:Because on our way to success, it may take much time. If we don’t have interest, we may give up.Il

In Turn 4, based on her judgment that success may take much time, Gu Lan drew a conclusion that people may give up and hence will not succeed if they lack interest in their work. There are seemingly two problems with Gu Lan’s inference. One is that success does not necessarily take time. It is not surprising to see some cases of instant success due to opportunities or sheer luck. The other problem with Gu Lan’s reasoning is that she was assuming that spending time on something entails interest. In other words, according to Gu Lan, if people do not have interest, they would not spend much time to pursue something and may give up prematurely. It is not necessarily true since people sometimes spend time on something out of obligation, a sense of duty, or even habit rather than interest. Therefore, Gu Lan’s inference in Turn 4 is at a low level.

### 5.5. Synthesis Exhibited in Student Talk

Synthesis is the ability to combine information, opinions, ideas, or arguments from diverse sources to create a new opinion, idea, or argument (Anderson and Krathwohl 2001; Ennis 1987).

The following excerpt is part of a whole-class discussion over the reasons for classroom reticence.

Excerpt 9
TurnSpeaker
Code1T:Do you want to share with us some of the reasons why some of us don’t like to talk in the classroom?
2Sun Miao: We don’t know well.
3T:We don’t know each other.
4Sun Miao: We are shy to talk with each other.
5T:OK. Good. This is a good reason. We don’t know each other. We are strangers. We don’t have a group. Especially for Chinese, we are not much extroverted. If we don’t know each other well, we are reluctant to talk.
6Chu Ying:It’s a culture.Sl7T:Culture.
8Chu Ying:We don’t have the habit.Sl9T: Habit. We don’t have the habit of sharing. Good.


In Turn 6, Chu Ying displayed her skill of synthesis by connecting the ideas given by the teacher in Turn 5 to generalize that a reluctance to speak up is culture-related. However, she did not elaborate on her synthesis so as to expose her reasoning process of combining the teacher’s ideas with her own to create a new argument. Although she made more contribution in Turn 8, she failed to add more substance to her argument of culture so as to elevate her synthesis to a higher level. In fact, her contribution in Turn 8 is a downgrading from ‘culture’ to ‘habit’ since ‘habit’ is at a lower level compared to culture. Thus, by saying ‘habit’, Chu Ying was displaying a low level of synthesis.

## 6. Discussion and Conclusions

The universally acknowledged value of critical thinking in both schools and workplaces has inspired relentless efforts from researchers and practitioners to develop tools of assessing critical thinking. Given the preponderance of quantitative instruments and researchers’ concerns over the quantitative assessment of critical thinking, this paper has proposed a coding scheme to facilitate a qualitative analysis of critical thinking exhibited in interaction. The coding scheme has been applied to some excerpts of authentic classroom dialogue to elaborate its meaning and illustrate its use.

The coding scheme consists of five categories of critical thinking skills, i.e., analysis, comparison, evaluation, inference, and synthesis, each of which is coded at low, medium and high levels. These skills align well with the high-order skills in Bloom’s taxonomy (Anderson and Krathwohl 2001), categorizing the goals that should be aimed at both in and outside the classrooms. In addition, similar to Bloom’s taxonomy, it is assumed that knowledge, whether in general or in specialized areas, is the indispensable precondition for these critical thinking skills to be applied in practice. Hence, the level of knowledge about the topic concerned should be taken into account in the use of this coding scheme since “good critical thinking is not possible without considerable prior knowledge of the issue or concept concerned” (Dinsmore and Fryer 2023, p. 19). That is to say, the absence of critical thinking is sometimes due to the shortage of relevant knowledge rather than inadequate critical thinking skills.

Although this coding scheme focuses on the skills involved in critical thinking without explicitly covering the disposition of critical thinking, such dispositions should be given due attention while using this coding scheme, as critical thinking has been operationalized as both a set of skills and dispositions (Bailin and Siegel 2003; Dunne 2018). In natural dialogues, the display of critical thinking is spontaneous since no prompts will be given to the participants of dialogue for the use of critical thinking. In other words, people should apply critical thinking skills, such as those covered in this coding scheme, on their own initiative rather than passively. This is in line with Kuhn’s (1999) argument that critical thinking is closely related to metacognitive competencies. That is, critical thinkers always regulate, reflect on, and adjust their thinking consciously. After they develop into an accomplished critical thinker, however, they would have deeply internalized these critical thinking skills so that their use of critical thinking skills is not just conscious but highly intuitive (Elder and Paul 1996).

Moreover, the cultural factor should be borne in mind when using this coding scheme. As our coding scheme is based on the normative context in which critical thinking is a western construct (Dinsmore and Fryer 2023), its conceptualization may be different in some non-Anglo-American cultures. Hence, this coding scheme may be adapted for the assessment of critical thinking in such cultures.

This research has several potential limitations to be noted. First, although our efforts are aimed at minimizing the one-size-fits-all limitations of standardized assessment in critical thinking (Rear 2019), this research has raised the concern of subjectivity as the illustration of this coding scheme is subject to the authors’ subjective interpretations. In order to avoid the potential bias incurred by such subjectivity, methods of triangulation should be adopted in future research. For example, the subjective interpretations of dialogue participants’ critical thinking can be member-checked by the participants to see whether the interpretations are faithful.

Second, we have not considered the influence of others when developing this coding scheme to evaluate a person’s critical thinking in dialogue. Since dialogue is a chain of interaction in which a turn builds on the previous turn (Bakhtin 1981), one participant’s dialogue acts will very likely influence the way other participants respond. Hence, some display of critical thinking skills may not be spontaneous but a result of others’ enlightenment. In future research, such a coding scheme may be improved by taking account of the degree to which a person’s display of critical thinking results from others’ dialogue acts.

Third, this coding scheme has not been empirically tested or validated. As the main purpose of this study is to propose a coding scheme for critical thinking, it has not assessed the reproducibility and applicability of the coding scheme. Future research may test the coding scheme by evaluating the inter-coder reliability, consulting expert panels, or comparing the coding results with those of a well-established quantitative assessment of critical thinking.

In spite of these limitations, the potential of this coding scheme is not confined to the assessment of critical thinking exhibited in the classroom dialogues. It can also be used to assess critical thinking in dialogues in everyday life in view of its resemblance with dialogues in the educational setting. All dialogues in a real sense emphasize reciprocity, that is, people involved in a dialogue take account of, react to, and add to each other’s contributions in order to deepen and develop the dialogue. In both the educational setting and real life, people engaged in dialogues interpret information, evaluate opinions, and express ideas. Communicators are also expected to display logic and reasoning in both settings. Such resemblance between classroom dialogues and dialogues in real life shows that this coding scheme is also applicable in the assessment of critical thinking displayed in everyday dialogues.

Hence, individuals can evaluate and reflect on their critical thinking skills by applying this coding scheme to dialogues in everyday life. By this means, they will increase their understanding of critical thinking, especially the different levels of critical thinking skills that can be used as a yardstick of analysis, comparison, evaluation, inference, and synthesis. Individuals can also practice applying these critical thinking skills to situations in everyday life to improve their judgment and facilitate their decision-making. In doing so, they are able to develop critical thinking habits and cultivate critical thinking dispositions.

Besides these critical thinking skills, people keen to become critical thinkers in everyday life should also improve themselves on several other aspects. First, they should try to expand their knowledge widely and develop themselves into experts in some areas so that their use of critical thinking skills can be substantively supported. Second, they should enhance their metacognitive awareness, for example, by monitoring and exerting control over their thinking process (Halpern 2014) and their mood, considering its impact on critical thinking (Lun et al. 2023). Third, they need to overcome the cultural constraints that may constitute a barrier to their critical thinking. This is especially true for people in east Asian countries deeply influenced by Confucianism, which attaches great importance to hierarchy and authority (Ziliotti 2022). People in these cultures should not be reluctant to be critical thinkers by realizing that thinking critically does not amount to having no respect for authority and therefore does not contradict their cultures.

## Figures and Tables

**Table 1:** Coding scheme for critical thinking.

Category	Code	Description
Analysis	Al	Students manage to break down information, issues, opinions, ideas, or arguments into their organic constituent elements or to identify/establish their relationships. But their analysis is not deep either because they treat the information, issues, opinions, ideas, or arguments superficially or because the information, issues, opinions, ideas or arguments are so simple that no sophisticated analysis is needed and hence manifested.
	Am	Students succeed in breaking down information, issues, opinions, ideas, or arguments into their organic constituent elements or to identify/establish their relationships. Their analysis is deeper than the low-level analysis, but it still has some problems.
	Ah	Students succeed in breaking down information, issues, opinions, ideas, or arguments into their organic constituent elements or identifying/establishing their relationships. And their analysis is deep and clearly expressed.
Comparison	Cl	Students manage to identify similarities or differences among different information, issues, opinions, ideas, or arguments. But their comparison is not deep either because they treat the information, issues, opinions, ideas or arguments superficially, or because the information, issues, opinions, ideas, or arguments are so simple that no sophisticated comparison is needed and hence manifested.
	Cm	Students succeed in identifying similarities or differences among different information, issues, opinions, ideas, or arguments. Their comparison is deeper than the low-level comparison, but it still has some problems.
	Ch	Students succeed in identifying similarities or differences among different information, issues, opinions, ideas, or arguments. And their comparison is deep and clearly expressed.
Evaluation	El	Students manage to judge the credibility, validity, value or significance of information, issues, opinions, ideas, or arguments. But their evaluation is not deep either because they treat the information, issues, opinions, ideas, or arguments superficially or because the information, issues, opinions, ideas, or arguments are so simple that no sophisticated evaluation is needed and hence manifested.
	Em	Students succeed in judging the credibility, validity, value or significance of information, issues, opinions, ideas, or arguments. Their evaluation is deeper than the low-level evaluation, but it still has some problems.
	Eh	Students succeed in judging the credibility, validity, value, or significance of information, issues, opinions, ideas, or arguments. And their evaluation is deep and clearly expressed.
Inference	Il	Students manage to draw logical conclusions from information, observation, experience, judgment, theory, or hypothesis. But their inference is not deep because they treat the information, observation, experience, judgment, theory, or hypothesis superficially.
	Im	Students succeed in drawing logical conclusions from information, observation, experience, judgment, theory, or hypothesis. Their inference is deeper than the low-level inference, but it still has some problems.
	Ih	Students succeed in drawing logical conclusions from information, observation, experience, judgment, theory, or hypothesis. And their inference is deep and clearly expressed.
Synthesis	Sl	Students manage to combine information, opinions, ideas, or arguments from diverse sources to create a new opinion, idea or argument. However, it is not deep either because they treat the information, opinions, ideas, or arguments superficially or because the information, opinions, ideas, or arguments are so simple that no sophisticated synthesis is needed and hence manifested.
	Sm	Students succeed in combining information, opinions, ideas, or arguments from diverse sources to create a new opinion, idea, or argument. Their synthesis is deeper than the low-level synthesis, but it still has some problems.
	Sh	Students succeed in combining information, opinions, ideas, or arguments from diverse sources to create a new opinion, idea, or argument. And their synthesis is deep and clearly expressed.

## Data Availability

The datasets presented in this study are not available due to ethical reasons.
